# An efficient grafting method for phytoplasma transmission in *Catharanthus roseus*

**DOI:** 10.1186/s13007-024-01139-w

**Published:** 2024-01-20

**Authors:** Ho-Chun Chang, Jen-Chih Chen

**Affiliations:** 1 Institute of Biotechnology, National Taiwan University, Taipei, 106 Taiwan, ROC; 2 Agricultural Biotechnology Research Center, Academia Sinica, Taipei, 106 Taiwan, ROC

**Keywords:** *Catharanthus roseus*, Grafting, Phytoplasma transmission, Phytoplasma symptom

## Abstract

**Background:**

Phytoplasmas are parasitic plant pathogens that reside intracellularly within the sieve tube cells. Phytoplasmas induce various symptoms, including floral virescence, phyllody, leaf yellowing, and witches’-broom. Currently, it is challenging to culture phytoplasma in vitro. In the laboratory, phytoplasmas are generally maintained in alternative host plants, such as *Catharanthus roseus*. Grafting is used to transmit phytoplasmas among the alternative hosts. During the experiment, scions from infected plants are grafted onto healthy plants using a side grafting method. However, the practice has certain limitations, including its inability to be applied to small plants and its irregular disease incidence.

**Results:**

Here, we demonstrate a new approach, penetration grafting, to overcome the limitations of side grafting. This grafting method allows phytoplasma to be efficiently and uniformly transmitted into the inoculated plants. No significant difference was observed in phytoplasma accumulation between both grafting techniques. However, penetration grafting allows rapid symptom development, saving waiting time and reducing space usage.

**Conclusions:**

This study provides a reliable and stable method for experiments that require grafting transmission.

## Background

Phytoplasmas are organisms with the smallest genome, with a diameter of only about 0.2–0.8 μm and a genome size of about 600–880 kb. Although it does not have a cell wall, it is classified into the Mollicutes class of Gram-positive bacteria based on the sequence similarity of 16 S rDNA (16 S ribosomal DNA). Phytoplasmas are important plant pathogens that can parasitize more than thousands of plant species. In plant hosts, they only exist in phloem sieve tubes. Their appearance is polymorphic because they do not have a cell wall [1–3]. Typical plant symptoms caused by phytoplasmas include virescence, phyllody, proliferation, witches’-broom, yellows, and dwarfism, causing severe harm to agricultural production [4]. In Taiwan, important crop diseases caused by phytoplasmas include Pear decline [5], peanut witches’-broom [6], sesame witches’-broom [7], and periwinkle leaf yellowing [8].

These diseases are mainly transmitted by phloem-sucking insects such as leafhoppers, planthoppers, and psyllids. When insects bite diseased plants, phytoplasmas proliferate in the salivary glands of the vector insects and then spread into the phloem of healthy plants by biting them [9]. In recent years, although researchers have finally isolated four phytoplasmas from grapes and cultured them in liquid and solid media to develop rapid and low-cost detection or effective plant protection agents [10], this method is still not popular, and the cultured bacteria rely on vector insects to transmit the bacteria to the target plants. The entire inoculation process still needs to be developed. In addition, phytoplasmas can also be transmitted across species through dodder (*Cuscuta austras*); this transmission method can spread phytoplasmas from infected plants to a healthy alternative host, *Catharanthus roseus*. After the disease occurs in *C. roseus*, it can be transmitted back to the healthy original host plant through dodder to induce the initial symptoms, thus confirming that the disease on the original infected plant is caused by a phytoplasma [11].

*Catharanthus roseus* is a perennial herbaceous plant of the genus *Catharanthus* in the Apocynaceae family. In addition to its horticultural ornamental value, it is also an important medicinal plant. Phytoplasma titers can accumulate very high in *C. roseus*, where the pathogen can be easily maintained. Therefore, it is suitable as an alternative host to preserve phytoplasmas from different plant hosts. It can be used as a model plant to study the pathogenesis of phytoplasma diseases and the interaction between phytoplasma and plants [12]. It was hypothesized that *C. roseus* could be an excellent host for bacteria that cannot be artificially cultured due to its high nutrient content inside the phloem. The phloem sap of *C. roseus* contains 63 compounds composed of 47.5% sugars, 27% organic acids, 12.1% amino acids, 8.8% sugar alcohols, 1.9% sugar acids, 1.8% fatty acids, and 0.7% unknown compounds. The phloem sap provides the nutrients required for the growth of these small-genome parasitic pathogens that cannot synthesize nucleotides, amino acids, or fatty acids on their own [13, 14].

Grafting, in addition to dodder, was also used to transmit the pathogen. Scions from 3-month-old *C. roseus* and grapes with phytoplasmas were heterologously grafted onto 2-week-old tomato rootstocks, and more than 80% of plants were successfully infected by phytoplasmas [15]. In the laboratory, most phytoplasmas are transmitted from the original host plants to *C. roseus* through dodders and then transmitted between *C. roseus* by grafting [12]. Therefore, the grafting technique plays a crucial role in bacterial transmission.

Grafting is an ancient horticultural technique, which mainly involves removing a part of a plant and connecting it to another plant so that the two parts grow together to form a new plant [16]. Standard grafting methods include approach grafting, whip grafting, cleft grafting, cut grafting, wedge grafting, side grafting, and budding grafting.

Traditionally, phytoplasmas are transmitted to healthy plants mainly through cleft grafting or lateral side grafting [17]. However, these methods can only be carried out after the plant used as the rootstock has grown for more than three months because the stems must reach 4–5 mm in diameter before processing. If the stems are too thin, it will be difficult to fix the diseased scion to the healthy recipient stably, and often, failures such as the scion’s death or accidental cutting of the rootstock may occur. In addition to time constraints, large *C. roseus* plants will occupy more space and make large-scale experiments challenging. In addition, using old plants often results in irregular symptom development and makes it challenging to study phytoplasma pathogenesis. This is especially true when virus-induced gene silencing (VIGS) is used to observe the onset and symptom development of phytoplasma diseases after the expression of a specific gene is suppressed because a large scale of plants will be needed in this type of experiment. Furthermore, the current VIGS experiment using Tobacco rattle virus (TRV) as a vector must be carried out at 22℃ [18]. This low temperature also slows down the growth of *C. roseus*. Therefore, it is necessary to develop an experimental method that can be applied to small plants with a high graft success rate, a consistent disease progression, and a stable phytoplasma concentration in each branch. However, this type of study is still lacking. Therefore, we developed a suitable method that can be applied to small plants and named it penetration grafting. The advantages of the newly developed method are discussed.

## Methods

### Plant materials and growing conditions

The F1 seeds of *Catharanthus roseus* cv. Titan were purchased from Yahemei Biotechnology. Seeds were germinated in the dark for 3 days at 22 ℃, and then seedlings were transferred to a growth chamber at 22 ℃ with light intensity at 80–100 µmol m^− 2^ s^− 1^ under 16 h of light/8 hours of darkness cycles. They were transferred to seedling trays seven days later and grew at 28 ℃ with the same light condition. After 4 weeks, seedlings were transferred to 4-inch pots. The *C. roseus* plants were prepared in 2 batches, with one collection growing for 129 days after sowing (large plants prepared for side grafting) and the other with a plant age of 63 days after sowing (small plants prepared for penetration grafting).

### Source and conservation of periwinkle leaf yellowing (PLY) phytoplasma

The PLY phytoplasma was collected from infected wild *C. roseus* plants in Dayuan District, Taoyuan City, Taiwan, in 2014; these plants had typical witches’ broom-like leaf clustering symptoms [19]. Side grafting was used to transmit phytoplasmas among *C. roseus* plants. Around 2 centimeters of twigs from PLY phytoplasma-infected plants were used as scions, and they were grafted onto the 6-week-old healthy *C. roseus* plants to preserve the phytoplasmas.

### Grafting methods for PLY phytoplasma transmission

**Fig. 1:**
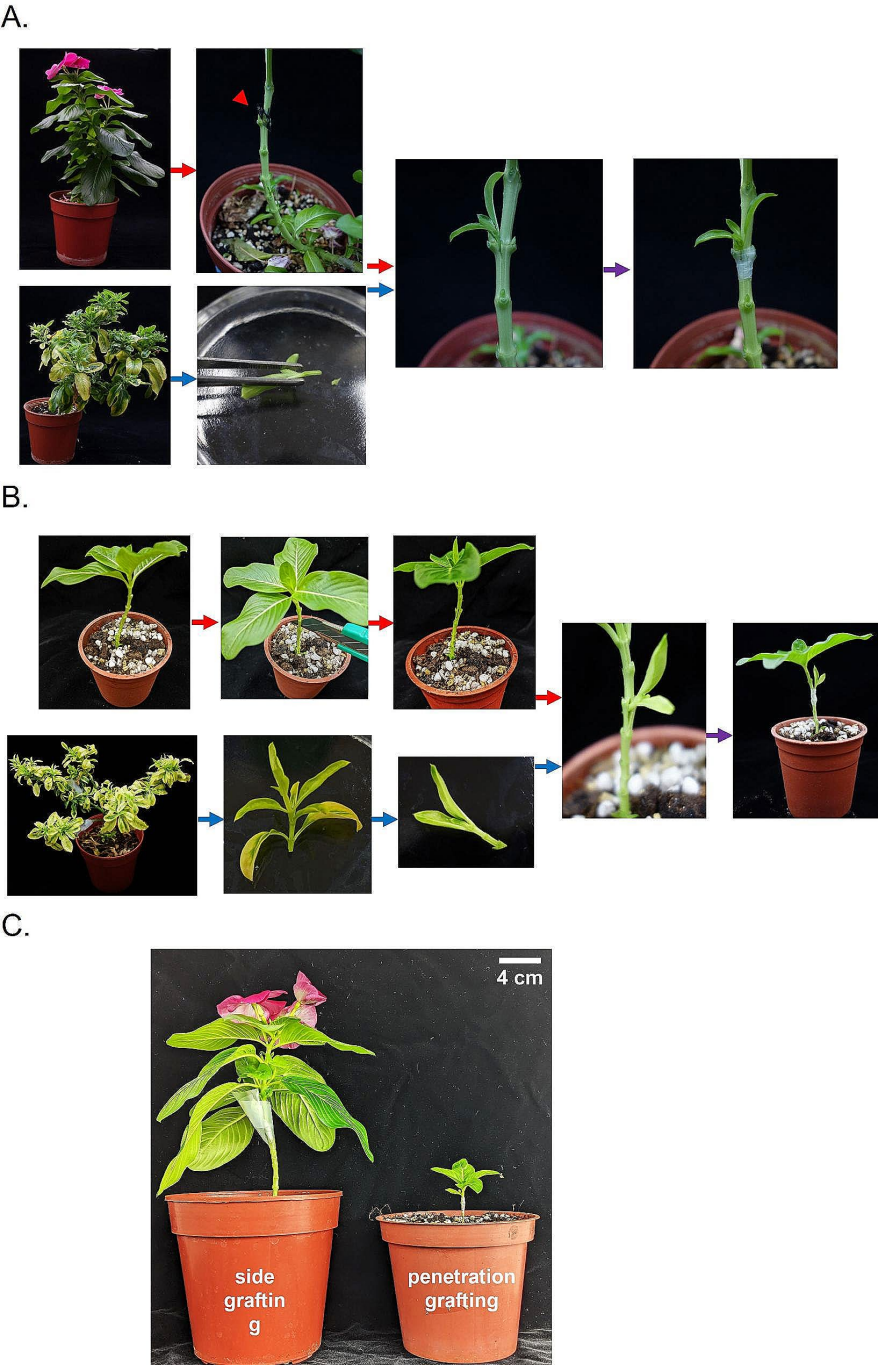
Diagram of grafting technique **(A)** The disease scions from phytoplasma-infected plants were cut into a ‘V’ shape and inserted into the slanting cut made on the healthy stock plants of periwinkle. The red arrowhead indicates the grafting site of the plant. **(B)** A box cutter is used to make a slit on the stem of the rootstock, and the infected scion cut into a 'V' shape is then inserted into the slit. **(C)** comparison of the completed grafting

The traditional side grafting method was used to transmit PLY phytoplasmas to *C. roseus* plants at 129 days of age [8]. The method is usually used when 90-day-old or older healthy *C. roseus* plants are used as the rootstock. First, the side branches and leaves were removed, and the top leaves and the terminal bud were left (defoliation). The stem was obliquely cut using a sharp razor blade near the vascular bundle of the branch to make a slender wedge for insertion of a diseased scion. A 2-centimeter-long twig from the top of a *C. roseus* plant that showed a typical withes’ broom symptom was cut, and its base was cut into a wedge shape to make the scion. The scion was then inserted into the slender wedge previously prepared and finally wrapped with paraffin tape (Fig. 1A).

The penetration grafting method was developed for phytoplasma transmission into tiny plants. A healthy *C. roseus* plant with a stem diameter of around 2 mm can be used as the rootstock. For the test, 63-day-old plants were used. The first defoliation step was similar to that in the traditional side grafting. The significant difference was to cut the middle of the stem using a razor blade to create a cleft. The preparation of phytoplasma-containing scions was again similar to the way used in side grafting. One scion was inserted into the cleft previously formed in the healthy rootstock and then wrapped with paraffin tape (Fig. 1B).

In this study, 129-day-old periwinkle plants were approximately 18 cm tall, while those at 63-day-old reached around 4 cm. Scions from the infected plants were separately grafted onto healthy periwinkle plants using side grafting for the 129-day-old plants and penetration grafting for the 63-day-old plants (Fig. 1C).

### Total DNA extraction

The extraction method was modified from Saqib [20]. Fifty mg of the leaves of *C. roseus* apexes whose disease symptoms reached the S3 stage were collected, frozen in liquid nitrogen, and ground into powders with a homogenizer; then, the homogenized sample was added with 0.5 ml CATB DNA extraction buffer (2% CTAB, 1.4 M NaCl, 20 mM EDTA, 100 mM Tris–HCl pH 8.0, 0.2% β-mercaptoethanol). The mixture was incubated at 65℃ for 30 min, then 0.25 ml of phenol:chloroform:isoamyl alcohol (25:24:1) was added into the mix and shaken at 100 rpm for 30 min and then centrifuged at 13,000 rpm for 15 min. The water phase of the sample was taken, and then 0.25 ml chloroform:isoamyl alcohol (24:1) was added to the water phase, shaken at 100 rpm for 30 min, and centrifuged at 13,000 rpm for 15 min. The upper phase (water phase) was then transferred to a new tube, and isopropanol was added to precipitate DNA at -20℃. The DNA was precipitated by centrifugation at 13,000 rpm for 10 min, and the DNA pellet was washed with 75% alcohol twice. Finally, the DNA was dissolved in water. DNA samples were quantified using Qubit™ fluorometer, Invitrogen Inc., USA, and stored at -20℃.

### Standard curve of PLY phytoplasma ***SecY*** gene

PLY phytoplasma concentration was determined using the *SecY* gene from the phytoplasma as the indicator. The primer set SecY-F/SecY-R (5’- GTGTTACAACAAGAGCAGCAATAA − 3’/5’- AAGACCAGGTGAACAAACTACTC − 3’) was used to perform absolute quantification real-time PCR on the total DNA with PLY phytoplasmas. To obtain the standard curve of the *SecY* gene, its fragment was amplified and purified, and the DNA concentration was measured using a Qubit™ fluorometer and then converted into a DNA copy number. The DNA fragment was diluted to 10^8^,10^7^,10^6^,10^5^,10^4^,10^3^,10^2^ copy/µl, and Real-time PCR was performed to determine the standard curve using Bio-Rad CFX384.

### Standard curve of the internal control gene, ***ubiquitin***

The *Ubiquitin* gene (*UBQ*) from the host plant, *C. roseus*, was used as the internal control for absolute quantification of PLY phytoplasmas. To establish the standard curve for real-time PCR of the *UBQ*, the total DNA of healthy *C. roseus* was serially diluted to 40 ng/µl, 10 ng/µl, 2.5 ng/µl, 0.625 ng/µl, 0.15625 ng/µl, 0.039063 ng/µl, 0.009766 ng/µl. The above 7 concentrations of Standard DNA were used as templates, and the primer set for the *UBQ* was Ubq-F/Ubq-R (5’-ACTCCATCTTGTCCCCGTCTCCG-3’/5’-ACAGAACACCACCACCGATACCCA-3’).

### Absolute quantification of real-time PCR

Bio-Rad CFX384 was used to perform the Real-time PCR analysis using the SYBR green method. The 2 x SYBR green pre-mix (Roche LightCycler® 480 SYBR Green I Master) was used, and the primer pairs used in the PCR were SecY-F/SecY-R and Ubq-F/Ubq-R. Three technical replicates were carried out for all samples. Real-time PCR reaction conditions are: (1) 95℃ for 2 min, (2) 95℃ for 30 s + 60℃ for 60 s, for 40 cycles, (3) after the cycle, the temperature is increased from 60℃ to 95℃ at a rate of 0.1 °C per second to obtain Melting curve of the product to detect whether non-specific products were produced.

### Statistical analysis

For the analysis of the grafting successful rate and incidence rate, the Chi-squared test was used to test whether there was a significant difference between the two grafting methods, and when the *P* < 0.05, it was considered significantly different. For symptom progression, the Chi-squared test was used to calculate the significant differences between the two inoculation methods, and when the *P* < 0.05, it was considered significantly different. For symptom progression, the ratio of different symptom stages was compared between two different inoculation methods, side-grafting and penetration-grafting. For the analysis of phytoplasma concentration, Excel was used to conduct t-test analysis to analyze and compare whether there were significant differences between treatments at the 5% significance level.

## Results

### Definition of PLY symptom stages

**Fig. 2:**
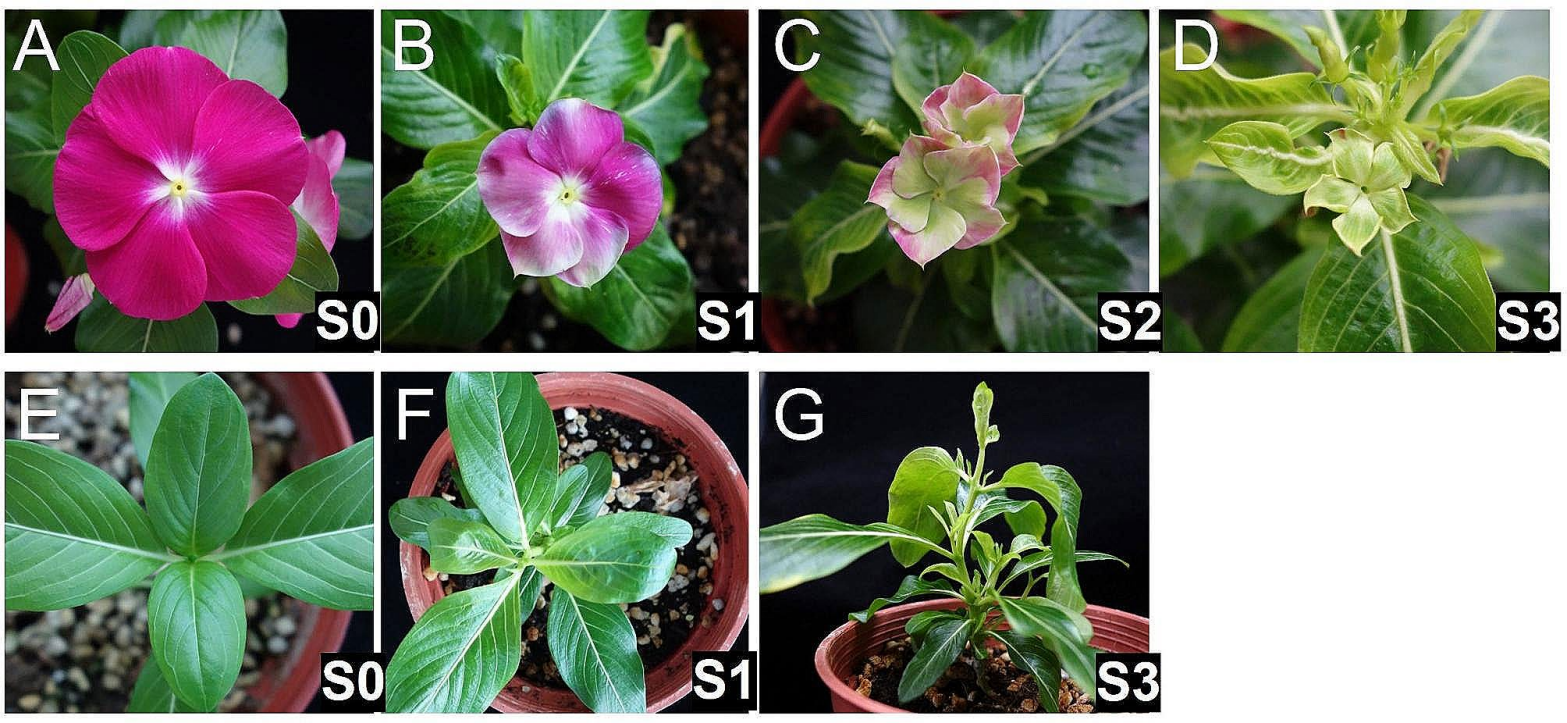
Floral and leaf symptoms of periwinkle leaf yellowing (PLY) phytoplasma-infected periwinkle plants **(A)** Flowers of healthy periwinkle; (**B** to **D**) flowers of PLY phytoplasma-infected periwinkle plants; **(E)** leaves of healthy periwinkle; (**F** and **G**) leaves or shoots of PLY phytoplasma-infected periwinkle plants. Flowers that exhibit discoloration or partial yellowing of leaves were defined as the S1 symptom stage (**B** and **F**). Flowers showing partial virescence are at the S2 stage **(C)**, while flowers that have completed virescence or shoot proliferation are at the S3 stage (**D** and **G**)

In the studies, PLY phytoplasma was used as the research pathogen. The definition of symptom stages according to the previous study was used to observe and record the onset date and symptom development of PLY phytoplasma-infected *C. roseus* plants [21]. The definition based on floral symptoms is according to different severity levels, in which S0 was defined as no visible symptoms (Fig. 2A); S1 is flowers showing discoloration (Fig. 2B); S2 is flowers showing partial virescence (Fig. 2C); and S3 flowers show virescence (Fig. 2D). Since young plants were used for the studies, the plants often did not enter the reproduction stage when infected, so floral symptoms were unsuitable for determining symptom severity. Therefore, we established severity levels for the vegetative stage based on symptom progression. They are S0: no visible symptoms (Fig. 2E); S1: leaf deformation and yellowing (Fig. 2F); and S3: severe proliferation (Fig. 2G). There was no S2 stage because the floral symptom reached S3 when the proliferation symptom occurred if large plants were used.

### Penetration grafting has a higher grafting success rate and incidence rate

To understand whether phytoplasma transmission through penetration-grafting can solve the problems caused by side-grafting, we compared the success rate of grafting and the incidence of phytoplasma disease in these two transmission methods. After grafting, we regarded the survival rate of scions as the success rate of grafting. The survival rate of scions using penetration grafting was as high as 92%; in comparison, using traditional side grafting, the survival rate of scions was only 75% (Table 1). The survival rate, however, was not statistically significant. We then compared the incidence rate of these two methods in cases of successful grafting. We found that the incidence rate was significantly higher when using penetration grafting than when using side grafting. The results showed that the incidence rate of plants grafted using the penetration grafting method was 100%, while the incidence rate of the grafted plants using the side grafting method was only 53% (Table 1). These results indicate that penetration grafting is a more stable and effective method to transmit phytoplasmas into healthy *C. roseus* plants.

**Table 1:** Summary of periwinkle grafting experiments after grafting for 32 days

Groups	Age^a^ (days)	success^b^/total grafts	Success rate (%)	symptomatic^c^/success grafts	Incidence rate (%)	
Side grafting	129	15/20	75%	8/15	53%	
Penetration grafting	63	23/25	92%^ns^	23/23	100%^**^	

Note: Chi-squared test was used to test whether there was a significant difference between the two grafting methods. ns: not significant, ** indicates *p value* < 0.01

^a^ the plant age when they were used for grafting experiments

^b^ the scion survived after grafting

^c^ the grafted plant exhibiting disease symptoms

### The onset time and symptom development are accelerated using penetration grafting

**Fig. 3:**
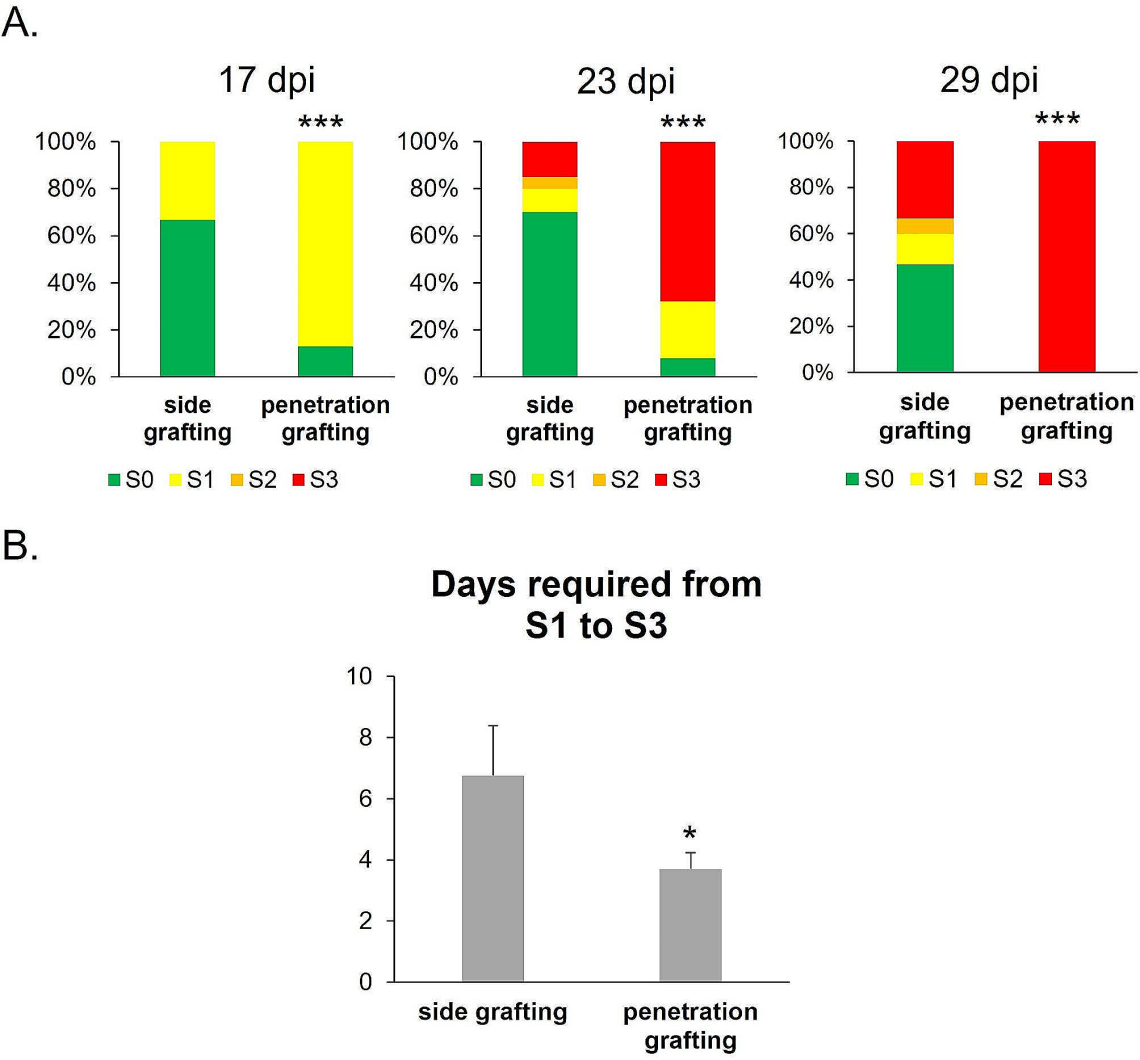
**(A)** Changes in the pathogenesis of PLY phytoplasma-infected periwinkle plants with different grafting methods. **(B)** Days required from S1 to S3. Each sample was determined from at least 6 biological replications. The symptom stages are defined according to Fig. 2; that are S0 (no visible symptom), S1 (flower discoloration or partial leaf yellowing), S2 (partial floral virescence), and S3 (completed floral virescence or shoot proliferation). The error bar indicates the SE of each set of replicates; * indicates *p value* < 0.05, and *** indicates *p value* < 0.001. dpi = days post-inoculation

In addition to the success rate of grafting and the incidence of the phytoplasma disease, we also explored whether these two grafting methods affected the onset time and symptom development of the disease in *C. roseus* plants. The results showed that 17 days after grafting, around 87% of the plants using the penetration-grafting for phytoplasma transmission had disease symptoms, and only 33% of the plants using the side-grafting method showed symptoms (Fig. 3A). The difference is statistically significant when the ratio of different symptom stages is compared. In addition, in terms of symptom development, the plants using side grafting for phytoplasma transmission went from S1 to S3 in around 7 days, while the plants using penetration-grafting for phytoplasma transmission shortened the development by approximately 3 days, that is the symptom from S1 to S3 took only 4 days (Fig. 3B).

There was no difference in phytoplasma accumulation between plants transmitted with phytoplasma through these two different methods.

**Fig. 4:**
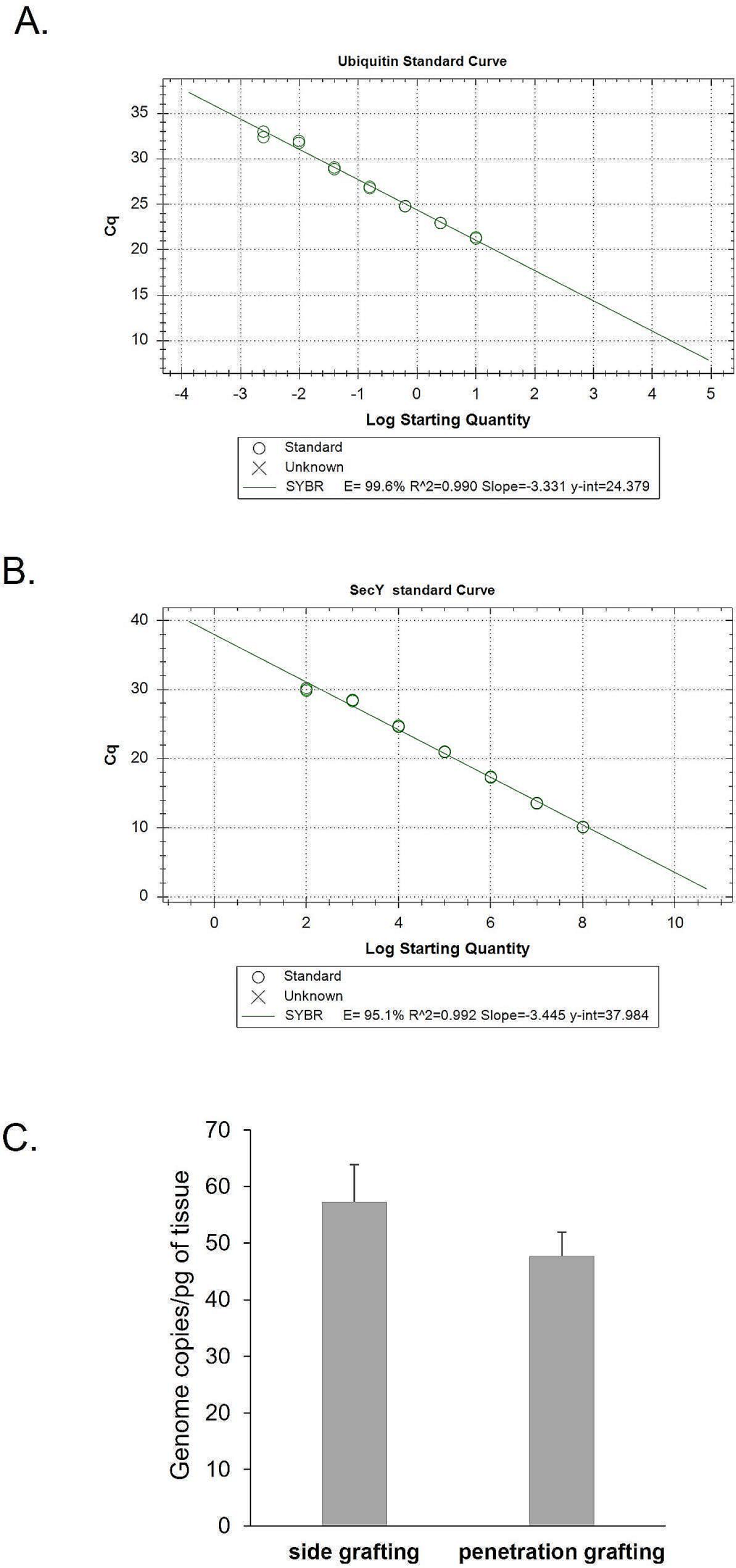
**(A)** Standard curve of real-time PCR Ct values of periwinkle ubiquitin gene. **(B)** Standard curve of real-time PCR Ct values of PLY phytoplasma *SecY*  gene. **(C)** The mean number of phytoplasma genome copies was measured in the apical leaves at the S3 stage (flowers that have completed virescence or plants showing shoot proliferation symptoms). Each group was determined from at least 6 biological replications. The error bar indicates the SE of each set of replicates

To understand whether the side-grafting and the penetration-grafting methods affected final phytoplasma accumulation, we took the first pair of leaves of the S3 stage plants. We performed absolute quantitative analysis through real-time PCR using the *UBQ* gene as the internal control for plant tissue content. The standard curve for the *UBQ* is shown in Fig. 4A. The standard curve for the phytoplasma *SecY* gene is shown in Fig. 4B. Experimental results show that the average phytoplasma accumulation in infected plants using side grafting was about 57.3 copies/pg plant tissue. The average phytoplasma accumulation in infected plants using penetration-grafting was 47.7 copies/pg plant tissue, about 20% less than the former, but there was no statistically significant difference. (Fig. 4C). These results show that no matter which grafting method was used, after successful transmission, phytoplasmas can accumulate in the terminal buds and cause changes in the flower shape and leaf shape.

## Discussion

Grafting is an ancient and common horticultural technique. It is a method of vegetative propagation of plants. It refers to the joining of two different plants into one individual. In the horticulture industry, disease-resistant or stress-resistant varieties are usually used as rootstocks, and varieties with high economic value are grafted to improve the growth potential of crops. As time passed, the grafting technique was used in many biological studies. For example, the discovery of flowering hormones can induce another plant to bloom by grafting a plant that matches the photoperiod [22]. In addition, grafting is also used to study plant-related pathogens, such as huanglongbing (HLB) caused by ‘*Candidatus* (*Ca.*) Liberibacter asiaticus’ [23], yellow leaf disease or leaf clustering caused by ‘*Ca.* Phytoplasma’ [12].

Regarding the research on phytoplasma transmission among plant hosts, most studies focus on the transmission through vector insects because this transmission mode is closer to the actual natural situation. When sugarcane was inoculated with SCWL phytoplasma by the leafhopper, *Matsumuratettix hiroglyphicus*, phytoplasmas first moved downward to the root and later spread systemically throughout the plant [24]. In contrast, when *Chrysanthemum coronarium* was inoculated with OY phytoplasma by leafhopper, *Macrosteles striifrons*, phytoplasmas were found first in the shoot apex and later found in the root [25]. However, since grafting is an essential technique for the propagation or production of horticultural crops, the spread of many phytoplasma diseases that have a significant impact on agriculture is also caused by grafting, such as pear decline in pear trees and jujube witches’-broom in jujube trees, and grapevine yellows in grapes [26, 27]. In addition, grafting is relatively simple to implement compared with vector insects and has a high inoculation success rate. The same batch of plants’ onset time and symptom progression are relatively stable. Therefore, in studying plant-phytoplasma interactions, transmission by grafting is more suitable than vector insects. Moreover, grafting does not require catching or raising insects, which requires specialized equipment [28]. Because of the relative stability of the grafting method, it has also been used to study the spread and distribution of phytoplasmas within the host plant and found that it took about 28 days for the phytoplasmas to be detected outside the scion. Phytoplasmas were found spreading toward the root first, and then they took 82 days to spread throughout the entire plant when phytoplasmas carrying twigs were grafted onto 1-year-old healthy *C. roseus* plants by side grafting [29]. In addition to side grafting, chip-budding grafting was used to inoculate apple proliferation (AP) phytoplasmas onto *C. roseus* plants. Around 90% of plants showed typical symptoms after the graft inoculation for two months [30]. The finding is similar to our results when using the side grafting method to inoculate PLY phytoplasmas in *C. roseus* plants. Around 33% of the successfully grafted plants showed the S1 symptom 17 days after inoculation, and approximately 50% of the plants exhibited various symptoms after 1 month of inoculation (Fig. 3). Finally (32 dpi), only 75% of the plants were successfully grafted, and 53% showed symptoms (Table 1). In comparison, 87% of the plants showed S1 symptoms 17 days after grafting, and 100% showed S3 symptoms one month later when the newly developed penetration-grafting method was used (Fig. 3). It can be seen from the results that the new approach has the advantages of a high grafting success rate and more consistent and rapid symptom progression. These advantages could be due to the smaller plants used for the method. The age-related susceptibility is an interesting phenomenon. Plant resistance to various pathogens is associated with plant developmental stages, and the phenomenon is called age-related resistance (ARR) [31, 32]. Generally, mature plants are less susceptible to pathogens than young plants [32]. Though ARR has not been discussed in *C. roseus*, it is ubiquitous in plants, and our finding provides a system for ARR studies in *C. roseus*. In terms of preparation of plants to be grafted, the plant size of *C. roseus* plants is usually more than 90 days old [33] because the stem of the plant needs to be at least about 4 ~ 5 mm to facilitate side grafting. The new method can carry out grafting experiments when the plant is 50–60 days old because the stem only needs to be 1 ~ 2 mm for the operation. Using small plants also significantly saves experimental time and space.

In addition to homologous grafting, phytoplasma transmission is also possible through heterologous grafting. In this regard, four grafting techniques, whip graft, bark graft, budding, and chip budding grafting, were used to introduce apple proliferation (AP) phytoplasmas into *C. roseus* plants. In this study, around 60% of the plants were successfully infected with AP phytoplasmas from infected apples to *C. roseus* plants. However, the latency phase of the disease was 4–6 months after grafting, significantly longer than graft transmission among *C. roseus* plants [30]. Because of the excellent phytoplasma inoculation performance shown by penetration grafting on *C. roseus* plants, although we have not yet conducted experiments on the phytoplasma transmission by heterologous grafting through penetration grafting, this method is worth trying. In particular, it would be nice if this method could introduce phytoplasmas into Solanaceae models, such as tomatoes and potatoes, that make it easier to conduct genetic and molecular biology studies [15].

Similar to the phenomenon observed in *C. roseus*, after a tomato was graft inoculated with phytoplasma ‘*Ca*. Phytoplasma solani’ phytoplasmas were first observed on the inoculated twigs and distributed into roots, shoot apex, and other side branches [28]. The accumulation of phytoplasmas in larger plants is prone to uneven distribution after inoculation. Moreover, non-symptomatic shoots can also be easily found when large *C. roseus* plants are used for graft inoculation [34]. On the other hand, penetration grafting, which can be applied on small plants with few side branches, systemically leads to infection of the entire plant rapidly and efficiently and potentially results in the even distribution of phytoplasmas. As for the phytoplasma concentration, the results also showed that although the concentration was slightly higher when the side grafting was used than when the penetration grafting was used, it was not statistically significant (Fig. 4C). Therefore, penetration grafting is suitable for studying phytoplasma accumulation and distribution.

## Conclusions

We have developed a convenient method for grafting and inoculating phytoplasmas on smaller plants. This method has a high grafting success rate, high incidence rate, rapid disease progression, and high consistency, so it is very suitable for use in studies of phytoplasma-plant interactions. Although we have only used the method in *C. roseus* plants, it should apply to other host plants. In addition to phytoplasmas, graft-transmitted or phloem-inhabiting pathogens, such as *Ca.* Liberibacter spp, *Ca.* Phlomobacter fragariae, and spiroplasmas, can also be transmitted through this method.

## Data Availability

All of the data and materials are available upon request.
